# ACSL4: biomarker, mediator and target in quadruple negative breast cancer

**DOI:** 10.18632/oncotarget.28453

**Published:** 2023-06-12

**Authors:** Marie E. Monaco

**Affiliations:** ^1^Department of Neuroscience and Physiology, NYU Grossman School of Medicine, New York, NY 10016, USA; ^2^VA NY Harbor Healthcare System, New York, NY 10010, USA

**Keywords:** ACSL4, breast cancer, ferroptosis, molecular subtype, quadruple negative breast cancer

## Abstract

Breast cancer is a heterogeneous disease for which effective treatment depends on correct categorization of its molecular subtype. For the last several decades this determination has relied on hormone receptor status for estrogen, progesterone and HER2. More recently, gene expression data have been generated that further stratify both receptor-positive and receptor-negative cancers. The fatty acid-activating enzyme, ACSL4, has been demonstrated to play a role in the malignant phenotype of a variety of cancers, including breast. This lipid metabolic enzyme is differentially expressed as a function of subtype in breast tumors, with highest expression observed in the mesenchymal (claudin low) and basal-like subtypes. Here we review data that support the potential of utilizing ACSL4 status as both a biomarker of molecular subtype and a predictor of response to a variety of targeted and non-targeted treatment regimens. Based on these findings, we suggest 3 expanded roles for ACSL4: 1. as a biomarker for classification of breast cancer subtypes; 2. as a predictor of sensitivity to hormone-based and certain other therapies; and 3. as a target for the development of new treatment modalities.

## INTRODUCTION

ACSL4 is one of five mammalian enzyme isoforms responsible for activation (thioesterification) of long-chain fatty acids as a prerequisite to their further utilization in lipid biosynthetic and fatty acid oxidative pathways [1]. ACSL4 is an extrinsic membrane protein that has been localized to mitochondria-associated membranes [2], peroxisomes [3] and lipid droplets [4]. It is most highly expressed in steroidogenic tissues [5] and has a substrate preference for highly unsaturated fatty acids, including arachidonic acid (AA), eicosapentaenoic acid and adrenic acid [6]. ACSL4 plays a pivotal role in AA metabolism. The conversion of AA to AA-CoA by ACSL4 is a mandatory step in the incorporation of AA into phospholipids and triglycerides, which can then function as structural membrane components (phospholipids), signal transducers (phosphinositides) and storage depots for AA (phospholipids and triglycerides). It is this stored AA that comprises the substrate for stimulated eicosanoid synthesis. Thus while ACSL4-mediated storage of AA initially lowers levels of unesterified AA, in the long term the increase in stored AA may result in enhanced eicosanoid production in response to stimulation of phospholipase activity [7].

ACSL4 activity has been implicated to play a role in both normal and abnormal physiology. Evidence to date suggests that ACSL4 functions in a variety of normal developmental and cell biological processes, including neuronal differentiation [8], germ line sex determination [9], adipocyte differentiation [10], insulin secretion [11], steroidogenesis [12, 13], and membrane fusion [14], as well as in a number of disease processes, including x-linked mental retardation [15], kidney disease [16], liver disease (NAFLD) [17], obesity [18], osteoarthritis [19], and a variety of cancers, including those of liver [20], colon [21], prostate [22, 23] and breast [22, 24, 25]. A recent review summarizes expression of ACSL4 in various cancers and its potential role as a target and biomarker [26].

A role for long-chain fatty acid activating enzymes in supporting the malignant phenotype was first suggested in 2000 when Cao et al. [27] asserted that ACSL4 could promote carcinogenesis by lowering the levels of unesterified AA, thus inhibiting AA-mediated apoptosis in cancer cells. Subsequently, Mashima et al. [28] suggested that ACSL activity, in general, functioned as a survival factor in cancer cells. For the most part, ACSL4 expression is upregulated in cancer when compared with normal tissue, as is observed in the instances of colorectal cancer [21, 29], hepatocellular carcinoma [30–32] and multiple myeloma [33] and has been demonstrated to mediate increases in proliferation, migration and invasion in these cancers. However, in breast cancer, ACSL4 mRNA expression is significantly less in cancer versus normal tissue. A recent meta-analysis of public databases comparing ACSL4 mRNA and protein expression in a variety of cancers confirmed that ACSL4 expression is decreased in malignant versus normal breast tissue, while being differentially expressed as a function of molecular subtype [34]. This is not surprising given the inverse relationship, described in detail below, between ACSL4 expression and receptor expression. In fact, most normal breast cells do not express estrogen receptor (ER) [35], while most breast cancers do express ER. Yet, regardless of whether ACSL4 mRNA expression is generally increased or decreased in malignant versus normal cells, ACSL4 expression is positively correlated with a more aggressive phenotype of that particular cancer [23–25]. With possible relevance to health disparities, and the increased incidence of triple negative breast cancer (TNBC) in the African American population, ACSL4 mRNA is overexpressed in the livers of African Americans [17], as is a variant of the AA synthetic enzyme, FADS1, thought to be responsible for the higher levels of both circulating and tissue AA present in the African American population [36]. These data suggest the possibility that increased AA levels may contribute to a biological basis for the increase in TNBC.

## ACSL4 IN BREAST CANCER

### Inverse relationship between ACSL4 expression and receptor expression/activity

Breast cancer is a heterologous disease initially subcategorized as a function of receptor expression with respect to ER, progesterone receptor (PR) and human epidermal growth factor 2 (HER2) receptors. Cancers lacking all three receptors are termed triple negative breast cancer (TNBC). TNBC exhibits a more aggressive phenotype than receptor positive cancer (RPBC) and currently lacks targeted treatment options. This receptor-based characterization has been further refined by means of mRNA expression analyses, with receptor-positive cancers comprising mainly the luminal A, luminal B and HER2-enriched subcategories [37], and most TNBC classified as basal-like [38]. TNBC has been further subdivided into 4 categories by Burstein et al. [39] based on mRNA expression data: luminal androgen receptor (LAR), mesenchymal (MES), basal-like immune-suppressed (BLIS) and basal-like immune-activated (BLIA). Lehmann et al. [40] have reported four similar subdivisions. Due to the fact that the LAR subcategory, which is unique in expressing androgen receptor (AR), is significantly different from the other three subcategories, AR-negative TNBCs have been grouped together in a category referred to as quadruple negative breast cancer (QNBC) [41–43]. As has previously been reported, ACSL4 overexpression is a defining characteristic of QNBC [24].

Observational studies have demonstrated that ACSL4 mRNA expression is inversely correlated with ER, AR and HER2 expression in both breast cancer cell lines and tumor samples, and is most highly expressed in TNBCs that fall into the category of claudin-low and basal-like cancers [24]. In particular, ACSL4 is expressed in TNBCs that lack AR, and it has been suggested that ACSL4 might function in general as a biomarker for this AR-negative class of TNBC (QNBC). Based on data derived from 71 breast cancer cell lines, positive ACSL4 expression predicted QNBC status with a sensitivity of 78% and a specificity of 86% [24]. mRNA expression data reported by Burstein et al. [39], which subcategorize TNBC into the 4 classes described above, confirm that ACSL4 mRNA is differentially expressed in TNBC as a function of AR status, with highest expression in mesenchymal breast cancers.

There are limited proteomic data with respect to breast cancer subtypes; however, these data also support the conclusion that ACSL4 is differentially overexpressed in basal-like breast cancers. A study comparing protein expression in luminal B versus basal-like breast cancer samples clearly indicates that ACSL4 protein is more highly expressed in the basal-like subset [44]. A more recent study supports this finding [45].

Experimental, as opposed to observational data, provide further evidence of the inverse relationship between ACSL4 and receptor expression. Table 1 summarizes the results from these experiments. Specifically, forced expression of ACSL4 in ER-positive, ACSL4-negative breast cancer cells lowers ER expression both *in vitro* and *in vivo*; and conversely, silencing of ER expression in MCF7 cells induces expression of ACSL4 *in vitro* [24]. Re-expression of ER in ACSL4-positive, ER-negative MDA-MB-231 cells has been reported to downregulate ACSL4 mRNA and protein expression [46]. When hybrid breast cancer cells are generated from ACSL4-positive, ER-negative MDA-MB-231 cells and ACSL4-negative, ER-positive ZR75-1 cells, mRNA expression profiles demonstrate that the hybrid cells most closely resemble the ACSL-4-positive MDA-MB-231 cells. Not only is ACSL4 expression preserved, but ER expression in the hybrid is ablated [47]. Of particular interest is the observation that forced expression of ACSL4 in estrogen dependent MCF7 cells induces resistance to the stimulatory effects of estrogen as well as to the inhibitory effects of tamoxifen, thus mediating resistance [24]. Conversely, subclones of MCF7 cells that have been selected for tamoxifen-resistance demonstrate an increase in ACSL4 mRNA expression (2.26-fold, *p* = 0.0013, [48]). Forced expression of ACSL4 in the HER2-enriched cell line, SKBr3, induces resistance to treatment with lapatinib [24]. That resistance to lapatinib is associated with ACSL4 expression is also supported by studies of lapatinib resistance in a variety of breast cancer cell lines [49, 50]. An analysis of the microarray data from these studies indicates that resistance to lapatinib is positively associated with expression of ACSL4.

**Table 1:** Experimental data supporting the inverse relationship between sex-steroid hormone receptor expression and ACSL4 expression

Experiment	Results	Reference GEO^*^ Number
Forced expression of ACSL4 in MCF7 cells *in vitro*	Increased ACSL4 Decreased ER, PR and AR	[24] GSE40968
Forced expression of ACSL4 in MCF7 cells *in vivo*	Increased ACSL4 Decreased ER and PR	[52]
Silencing of ESR1 in MCF7 *in vitro*	Increased ACSL4 Decreased ER	[24] GDS4061
Forced expression of Raf-1 in MCF7 cells *in vitro*	Increased ACSL4 Decreased ER	[22] GDS1925
Hybrid cells derived from ACSL4-positive, ER-negative MDA-MB-231 and ACSL4-negative, ER-positive ZR75-1	Hybrid cells are ACSL4-positive and ER-negative	[47] GDS4067
Forced expression of ACSL4 in HER2-positive SKBr3 cells	Decreased response to treatment with lapatinib	[24] GSE40968

^*^GEO: Gene Expression Omnibus (https://www.ncbi.nlm.nih.gov/geo/).

This inverse relationship between ACSL4 and hormone receptor expression is not unique to breast cancer. Similar results have been reported for the relationship between AR and ACSL4 expression in prostate cancer. LNCaP androgen-dependent prostate cancer cells, which do not express ACSL4, induce expression of ACSL4 when transformed into LNCaP-AI, which is androgen independent [22]. Additional studies expand on a role for ACSL4 in promoting prostate cancer growth, invasion and hormone resistance, confirming an inverse relationship between ACSL4 expression and that of the androgen receptor [23]. As observed for breast cancer, the presence of both ACSL4 and androgen receptor in prostate cancer cells predicts resistance to androgen deprivation therapies.

In summary, ACSL4 expression is inversely correlated with ER, AR and HER2 expression. Forced expression of ACSL4 in cells that express these receptors induces a decrease in receptor expression and resistance to receptor-based therapies. As such, ACSL4 status could function as a biomarker of resistance to hormonal therapy. Based on the fact that ACSL4 is more highly expressed in the more aggressive breast cancer subtypes, it might be expected that ACSL4 status would also function as a prognostic indicator of disease progression and overall survival. However, data with respect the prognostic significance of ACSL4 expression are mixed, most likely due to a number of confounding parameters, such as the particular cutoff used to differentiate high from low expression, the follow-up threshold, receptor status and previous treatment [34]. For example, while the expression of ACSL4 coincides with a more aggressive subtype of breast cancer and thus might be assumed to be associated with a worse prognosis, it is conceivable that the increased proliferation associated with ACSL4 expression might make cancer cells more sensitive to certain chemotherapeutic interventions and thus be associated with a better prognosis. Further analyses will be required to determine the role of ACSL4 as a prognostic biomarker.

### ACSL4 expression induces an aggressive phenotype: induction of EMT and stimulation of proliferation, migration and invasion

The epithelial-to-mesenchymal transition (EMT) is a process by which epithelial cells take on the characteristics of mesenchymal cells with respect to migration and invasion thus increasing their ability to metastasize [51]. Experiments designed to either induce expression of ACSL4 in ACSL4-negative cells (MCF7 or SKBr3) or ablate expression in ACSL4-positive cells (MDA-MB-231) demonstrate a role for ACSL4 in stimulating proliferation, migration and invasion in breast cancer [24, 25, 52]. Similar results are observed in prostate cancer cells; and in colon cancer cells, ACSL4 has been shown to be part of a triad (ACL1/ACSL4/SCD) that induces EMT [29, 53].

Data also support a role for ACSL4 in mediating EMT induced by other agents. RAF1, for example, which has been linked to EMT [54], induces expression of ACSL4 mRNA when overexpressed in MCF7 cells [24]. Similar results are seen for SNAI1 induction of EMT in MCF7 cells [55]. Analysis of microarray data indicates that the tyrosine phosphatase, SHP2, which has been demonstrated to increase migration of TNBC cells [56] and to have a role in promoting TNBC and basal-like breast cancer [57], and whose inhibition results in a basal-to-luminal transition [58], regulates expression of ACSL4 protein in MA-10 Leydig cells [59]. The transcription factor, FOXM1, has been demonstrated to effect breast cancer cell migration; and functional assays in MDA-MB-231 mesenchymal breast cancer cells implicate ACSL4 as possible mediator of the FOXM1 effect [60]. Conversely, data support a role for FOXM1 in mediating ACSL4-induced radioresistance in breast cancer cells [61]. Data also suggest that ACSL4 plays a role in conferring susceptibility to breast cancer tumorigenesis associated with the expression of the PADI2 gene [62].

### Mechanism of ACSL4 action

**Figure 1:**
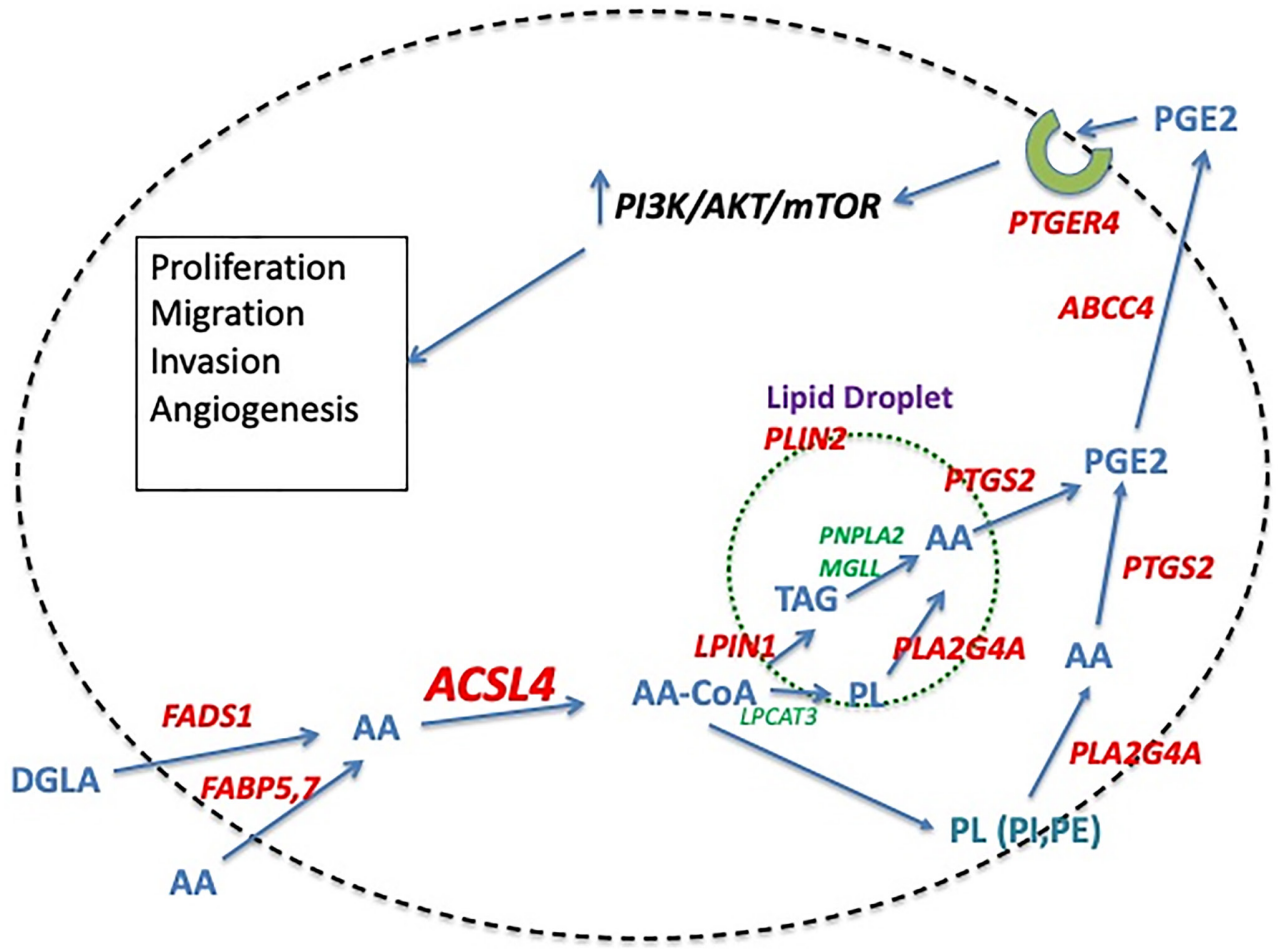
Proposed pathway of ACSL4-mediated stimulation of QNBC cells. The model outlines a pathway that ultimately results in the increased growth and survival of breast cancer cells as the result of increased production of PGE2. ACSL4 activity is hypothesized to contribute to the increase in PGE2 by increasing the amount of AA stored in phospholipids and triglycerides that is subsequently available for conversion to PGE2 upon stimulation of phospholipase activity. Protein entities in RED are either overexpressed in QNBC versus receptor-positive breast cancer (RPBC), or activated as a result of ACSL4 expression, while those in GREEN are either underexpressed or similarly expressed in QNBC versus RPBC. Abbreviations: AA: arachidonic acid; ABCC4: ATP binding cassette subfamily C member 4; ACSL4: Acyl-CoA synthetase long chain family member 4; AKT: protein kinase B; DGLA: Dihomo-γ-linolenic acid; FABP5,7: fatty acid binding protein 5, 7; FADS1: fatty acid desaturase 1; LPCAT3: lysophosphatidylcholine acyltransferase 3; LPIN1: lipin1; MGLL: monoacylglycerol lipase; mTOR: mammalian target of rapamycin; PC: phosphatidylcholine; PE: phosphatidylethanolamine; PGE2: prostaglandin E2; PI3K: phosphoinositide-3-kinase; PL: phospholipid; PLA2G4: phospholipase A2 group IVa; PLIN2: perilipin 2; PTGER4: prostaglandin E receptor 4; PTGS2: prostaglandin endoperoxide synthase 2; TAG: triacylglycerol.

Data presented above support a role for ACSL4 activity in inducing a more aggressive form of breast cancer. The question remains as to the mechanism of the ACSL4 effect. How does activation of specific long-chain fatty acids by ACSL4 instigate these changes? Figure 1 outlines a potential pathway for ACSL4-induced effects on breast cancer cells that relies on enhanced production of PGE2 to mediate effects on growth and metastasis. Elevated PGE2 levels have been shown to be associated with more aggressive breast cancer phenotypes [63]. The preference of ACSL4 for AA as a substrate suggests that the actions of ACSL4 may be mediated, at least in part, by elements of the AA metabolic pathway that terminate in the production of eicosanoids. Data reported by Maloberti et al. [25] support this hypothesis. These data demonstrate that forced expression of ACSL4 in MCF7 cells increases both the level of PTGS2 (COX2) protein as well as the production of the prostaglandin, PGE2. PGE2, in turn, has been demonstrated to induce EMT and concomitant aggressive behaviors in a variety of cancers, including breast [64]. In brief, the hypothesis entails a sequence of events whereby the conversion of free AA to AA-CoA facilitated by ACSL4 increases the level of stored AA in the form of phospholipids and triglycerides, which are then available upon stimulation of phospholipase activity to be utilized for the production of PGE2. This PGE2, in turn, can act in an autocrine or paracrine manner to induce increased proliferation, migration, invasion and angiogenesis. A role for mTOR in ACSL4-mediated effects on growth and survival is supported by the findings of Orlando et al. [65]. This same study also noted an increase in AKT (protein kinase B or PKB) phosphorylation on Ser473.

**Figure 2:**
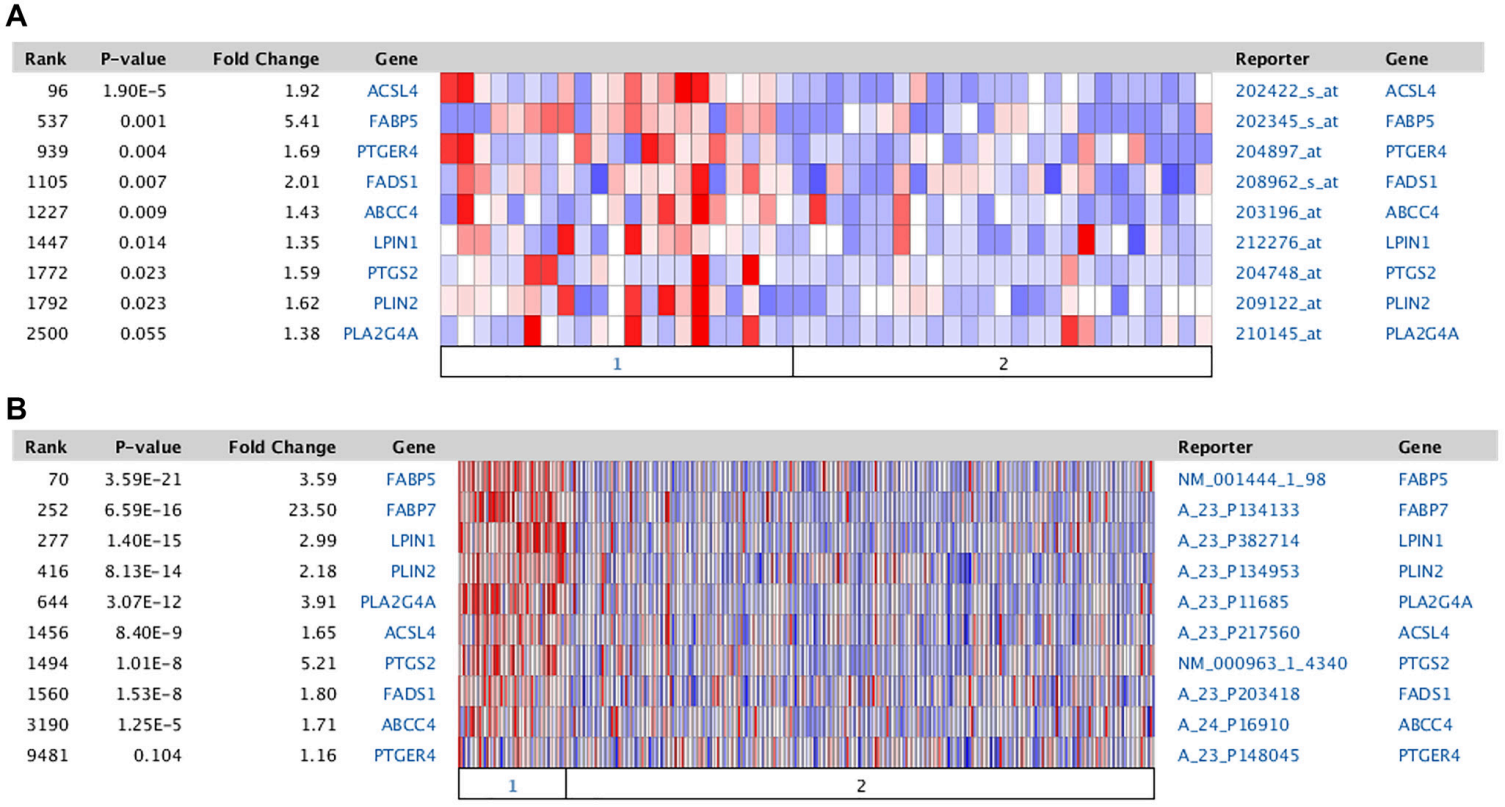
Comparison of the expression of Arachidonic acid metabolic genes between TNBC and RPBC. Data from Oncomine [68]. The Oncomine™ platform (ThermoFisher, Inc., Ann Arbor, MI) was used for analysis and visualization. (**A**) Samples derived from breast cancer cell lines [69]. Section 1: TNBC, 21 samples; section 2: RPBC, 25 samples. (**B**) Data from The Cancer Genome Atlas (TCGA). Section 1: TNBC, 46 samples; section 2: RPBC, 250 samples. Values represent log 2 median-centered intensity. Blue: least expressed genes; red: most expressed. Grey: not measured. Abbreviations as in Figure 1.

Further supporting the hypothesis outlined in Figure 1 is the concomitant increase in mRNA expression of other protein entities comprising the AA metabolic pathway. The genes involved in the metabolism of AA are broken down into 6 categories: Synthesis of AA from the precursor, DGLA (FADS1); Transport of AA into the cell (FABP5); Activation of AA (ACSL4); Storage of AA (PLIN2, LPIN1); Mobilization of AA (MGLL, PNPLA2, PLA2G4A); and Utilization of AA in the synthesis of PGE2 and its action via the PTGER4 receptor (PTGS2, ABCC4, PTGER4). A previous analysis indicated that there is increased mRNA expression of a number of these proteins in TNBC [66]. The details from two specific studies are documented as heat maps in Figure 2. These data indicate similar results for both cell lines (Figure 2A) and tumor samples (Figure 2B). Table 2A details the results of an ACSL4 co-expression analysis for some of these genes. Only those results with a Spearman coefficient >0.4 are included. Note that alterations in mRNA expression patterns may not reflect changes in protein expression or enzyme activity. Although proteomic analyses are limited, the available data align with the mRNA expression data, as demonstrated in Table 2B comparing tumor samples from luminal B and basal-like breast cancers [44]. For each protein listed in the table, expression was higher in the basal-like sample than in the luminal B sample. In addition, forced expression of ACSL4 in MCF7 cells has been demonstrated to induce an increase in PTGS2 protein [25]. In summary, both mRNA and protein expression data, where available, align with the hypothesis presented in Figure 1, suggesting that breast cancer molecular subtypes that express ACSL4 also exhibit increased expression of other protein entities involved in AA metabolism.

**Table 2A:** ACSL4 co-expression analysis in breast cancer samples

Correlated Gene	Spearman’s Coefficient	*p-*Value	*q-*Value
**FABP5**	0.454	1.05e-51	1.71e-50
**PLIN2**	0.466	1.22e-54	2.21e-53
**LPIN1**	0.448	3.85e-50	5.87e-49
**PLA2G4A**	0.740	5.27e-173	7.10e-170
**PTGS2**	0.574	3.11e-88	2.02e-86
**ABCC4**	0.634	1.20e-112	1.95e-110
**PTGER4**	0.627	7.04e-110	1.03e-107

Data from TCGA Pan Cancer Atlas, *n* = 994 [67], as reported on http://www.cbioportal.org/. Samples are comprised of invasive breast cancers.

**Table 2B:** AA metabolic protein expression in basal-like and luminal B breast cancer subtypes

GENE	BL	LB	*p-*Value	RANK
**LPL**	0.03	−3.52	0.024	903
**FABP5**	−0.05	−6.80	0.0008	168
**ACSL4**	−1.51	−4.95	0.011	653
**PLIN2**	0.34	−3.88	0.0002	80
**LPIN1**	−0.20	−1.84	0.023	884
**PLA2G4A**	−1.04	−3.93	0.013	713
**PTGS2**	−2.08	−4.27	0.123	1897

Data taken from Huang et al. [44] are an iTRAQ-based proteomic quantification analysis of the expression ratios of 12,794 proteins in patient-derived xenograft samples.

Lastly, it has been previously demonstrated that silencing of the estrogen receptor alpha gene (ESR1) in MCF7 cells induces expression of ACSL4 mRNA [24], raising the question of whether such silencing affects the expression of any other genes in the AA pathway. Table 3 illustrates the effect of ESR1 silencing of mRNA expression of the AA metabolic pathway genes in MCF7 cells. As expected from data documenting an inverse relationship between ESR1 and ACSL4 expression (Table 1), silencing of ESR1 expression in MCF7 cells results in significant increases in mRNA expression of genes involved in PGE2 synthesis and action. No significant differences were observed for mRNA levels of ALOX enzymes involved in leukotriene production.

**Table 3:** Effect of silencing ESR1 on expression of AA metabolic pathway genes in MCF7 cells

GENE	Control	ESR1-Silenced	Fold Change	*p-*Value
**FABP5**	1566 ± 54	4101 ± 269	2.62	8.94e-05
**FABP7**	NE	NE		
**FADS1**	298 ± 14	1597 ± 152	5.36	1.20e-04
**ACSL4**	155 ± 10	1960 ± 75	12.6	1.99e-06
**PLIN2**	95 ± 10	576 ± 87	6.06	3.40e-04
**PLA2G4A**	10 ± 0	128 ± 8	12.8	1.17e-05
**PTGS2**	12 ± 2	81 ± 32	6.75	0.019
**ABCC4**	211 ± 3	423 ± 36	2.00	5.60e-04
**PTGER4**	201 ± 12	403 ÷ 103	2.00	0.022
**ALOX5**	25 ± 1	25 ± 3	0	0.958
**ALOX12**	29 ± 2	25 ± 1	−1.16	0.047
**ALOX15**	221 ± 3	27 ÷ 3	−8.18	1.44e-07
**ESR1**	4779 ± 66	21 ± 1	−250	2.86e-08
**PGR**	382 ± 7	25 ± 3	−15.4	1.49e-07
**AR**	903 ± 68	36 ± 1	−25.6	2.51e-05

Data shown are mRNA expression values in arbitrary units. Abbreviations are as described for Figure 1 plus ALOX: arachidonate lipoxygenase; ESR1: estrogen receptor alpha; PGR: progesterone receptor; AR: androgen receptor; NE: not expressed. Calculations were made from array data reported by Al Saleh et al. [70]. Values shown represent the mean ± 1 SD of three separate data points.

### ACSL4 is a biomarker for sensitivity to some chemotherapeutic reagents

In addition to inducing resistance to hormone-based therapies, ACSL4 expression is also associated with, and can mediate, resistance to chemotherapeutic regimens. Forced expression of ACSL4 in breast cancer cells induces resistance to the cytotoxic effects of etoposide [24], cisplatin, doxorubicin and paclitaxel, possibly mediated by an increased expression of multidrug resistance transporters including ABCC4, ABCC8 and ABCG2 [71]. The data indicate that ACSL4 inhibits drug accumulation in cells by increasing efflux. Another possible mechanism for ACSL4-mediated resistance to chemotherapy is the role it plays in the formation of lipid droplets [4], which have been shown to be associated with increased chemoresistance [72]. Thus, inhibition of ACSL4 activity in chemotherapy resistant tumors might be an effective treatment strategy, as well as serving as a biomarker of resistance.

### ACSL4 is a mediator of ferroptosis

Ferroptosis is a newly described, iron-dependent mechanism of non-apoptotic cell death mediated by peroxidation of phosphatidylethanolamine-associated AA and adrenic acid [73]. Incorporation of these fatty acid moieties into phospholipids requires activation by the enzyme, ACSL4. Cells lacking ACSL4 activity are unable to undergo classical ferroptosis. Evidence to date suggests that ferroptotic cell death plays a role in normal development as well as in a variety of pathological states (for review see [74]). Inhibition of ACSL4 in these pathological states might mitigate associated morbidities and mortality. On the other hand, in some instances, such as cancer, activation of ferroptosis might comprise a viable treatment option, in which case expression of ACSL4 would be a requirement for sensitivity to ferroptosis-inducing reagents. This has, in fact, been demonstrated to be the case with respect to the response of human breast cancer cell lines to ferroptotic reagents [75]. Only those cells that express ACSL4 (basal-like, receptor-negative cell lines) were found to be sensitive to ferroptosis. Similar results have been reported for cell lines derived from a variety of other cancers [76]. More recently, ACSL4 has been shown to mediate immunogenic tumor ferroptosis induced by cytotoxic T lymphocytes via IFNγ [77]. In fact, ACSL4 expression has also been suggested to be positively correlated with immune infiltration in breast cancer [34]. Thus expression of ACSL4 has been described as a “double-edged” sword in multiple myeloma since it both increases tumor progression and is a requirement for induction of ferroptosis [33]. Recently, resistance to induction of ferroptosis in a pancreatic cancer model has been attributed to the secretion of exosomes by cancer-associated fibroblasts that contain an miRNA (miR-3173-5p) that targets and down-regulates ACSL4 [78]. In fact, a number of studies have demonstrated that ACSL4 expression is regulated by a variety of miRNA species in several cancers, including hepatocellular [79], colorectal [53] and ovarian [80].

### ACSL4 is a potential target in cancer treatment

Targeting ACSL4 in cancer treatment, whether it involves forced expression or inhibition, will most likely not comprise a lone therapy but will more likely function as part of a combined therapy. First, it is clear that cells, including malignant cells, are not completely dependent on ACSL4 for fatty acid activation. A number of both normal and cancerous cell types do not express ACSL4 and rely on the alternate isoforms (ACSL1, 3, 5 and/or 6) to carry out activation functions. Preclinical data clearly demonstrate that effects of ACSL4 on proliferation, migration and invasion of cancer cells are incremental rather than absolute [24, 25, 52]. Thus while inhibiting fatty acid activation has the distinct advantage of blocking the utilization of fatty acids from both exogenous and endogenous (*de novo* synthesis) sources, it will likely require inhibition of several isoforms simultaneously to be most effective. Preclinical studies have demonstrated the increased efficacy of targeting ACSL4 in combination with targeting other potential oncogenic factors such as mTOR [81] and lipoxygenases [52]. A recent report describes the development of a small molecule inhibitor of ACSL4, PRGL493 (N-(4-(3-(5-methylfuran-2-yl)-1-phenyl-1H-pyrazol-4-yl)-3,4-dihydrobenzo[4,5]imidazo[1,2-a][1,3,5]triazin-2yl)acetamide) that blocks cell proliferation and tumor growth in both breast and prostate cancer models as well as sensitizing tumor cells to chemotherapeutic and hormonal treatment [82]. This compound was shown to inhibit recombinant ACSL4 enzymatic activity *in vitro* as well as conversion of AA to arachidonoyl-CoA in cell cultures. It fails to inhibit proliferation of MCF7 cells lacking ACSL4 expression, suggesting specificity for ACSL4.

## DISCUSSION

Breast cancers comprise a heterogeneous disease that has routinely been categorized by receptor status with respect to estrogen, progesterone, HER2 and more recently androgen. Cancers lacking the first three receptors are classified as triple negative breast cancer (TNBC), while those lacking all four, quadruple negative breast cancer (QNBC). Treatments have been developed which specifically target these receptors; however, receptor-negative breast cancers are more aggressive and, to date, lack targeted therapies. Recently, TNBC has been subcategorized into four subtypes based on gene expression data: luminal androgen receptor (LAR), mesenchymal (MES), basal-like immune-suppressed (BLIS) and basal-like immune-activated (BLIA) [38]. The data summarized in this review suggest that a lipid metabolic enzyme, ACSL4, responsible for the activation of long-chain polyunsaturated fatty acids as a prerequisite to both incorporation into complex lipids as well as oxidation, functions as a biomarker for receptor negative status (QNBC) as well as for resistance to hormone-based therapies in receptor positive cancers. As such, we postulate that measurement of ACSL4 protein expression in breast cancer tissue would differentiate between receptor-positive and receptor-negative cancers, as well as predict which receptor-positive cancers might be resistant to hormonal therapies. Thus a single assay might identify a class of cancers, namely QNBC, that would ordinarily require four separate assays. This hypothesis has been tested in breast cancer cell lines where positive ACSL4 protein expression predicted QNBC status with a sensitivity of 78% and a specificity of 86% [24]. Similar studies have yet to be undertaken in tumor samples.

Additional data indicate that ACSL4 expression is associated with increased proliferation, migration and invasion of breast cancer cells both *in vitro* and *in vivo*. As such, ACSL4 presents a possible target for treatment, and indeed the search for specific inhibitors is underway, with one promising candidate recently reported. Furthermore, it is possible that successful inhibition of ACSL4 in breast cancer cells might render these cells more sensitive to other treatments, both hormone-based as well as non-specific chemotherapeutic regimens.

Many cancers, including breast, are dependent on *de novo* fatty acid synthesis and as a result there has been a concerted effort to capitalize on this dependence by developing inhibitors of fatty acid synthetase (FASN). However, these attempts have yet to translate to the clinic due to issues involving lack of specificity and unacceptable toxicity [83]. Interference with lipid metabolism *via* blockade of ACSL4 might have advantages over a more general inhibition of *de novo* fatty acid synthesis in that there are four other long-chain fatty acid activating enzymes that would continue to function.

Since ACSL4 has been demonstrated to play a role in augmenting synthesis of the inflammatory prostaglandin, PGE2, it may be possible to ameliorate its effects on proliferation, migration and invasion in QNBC by blocking the action of PGE2 at the level of its receptor, PTGER4. Such inhibitors already exist and it has been suggested that they might be effective in treating colon cancer [84]. It is conceivable that those breast cancers expressing high levels of ACSL4 might be particularly sensitive to such inhibition.

Due to the role of ACSL4 in supporting sex steroid biosynthesis in adrenal and gonadal tissue, inhibition of ACSL4 activity may enhance the effects of treatments aimed at lowering endogenous levels/activity of these hormones.

Finally, the central role of ACSL4 activity in mediating ferroptosis offers an additional, compelling rationale for determining the status of its expression in breast as well as other cancers, since only those cancers positive for its expression are sensitive to induction of classical ferroptosis as a possible treatment modality. It is conceivable that in the future many different types of malignancies will routinely be assessed for expression of ACSL4 as a prerequisite to treatment with ferroptosis-inducing reagents.

To date, initiatives aimed at the development of ACSL4 as a biomarker for classification of molecular subtype or as the basis for treatment have been lacking. While gene expression data are available, protein expression data are limited. It will be mandatory to generate such data to verify the utility of ACSL4 measurement as both a biomarker and treatment target. With respect to breast cancer, expression of ACSL4 protein would strongly suggest insensitivity to receptor-targeted treatment while indicating potential sensitivity to induced ferroptosis. Inhibitors of ACSL4 activity could prove useful in slowing proliferation of ACSL4-positive cancers. While the use of receptor biomarkers in the diagnosis and treatment of breast cancer has proved extraordinarily useful, perhaps it is time to consider adding routine measurement of ACSL4 protein as a predictive indicator in the management of breast cancer.

## CONCLUSIONS

ACSL4 has been demonstrated to play a pivotal role in both normal physiology as well as in a variety of disease states, including breast and other cancers. Data support a potential role for this protein as a single biomarker for classification of breast cancer subtypes, since its expression is indicative of a negative receptor status/resistance to receptor-dependent treatments with respect to ER, PR, AR and HER2 in breast cancer. ACSL4 status has also been demonstrated to predict response to a variety of chemotherapeutic reagents as well as to ferroptotic reagents. The ability of ACSL4 to induce a more aggressive phenotype suggests it might be a potential target for inhibition in the treatment of breast cancer. Additional studies are needed to demonstrate the utility of this protein as both a biomarker and target in the classification and treatment of breast cancer. In particular, an assessment of ACSL4 protein expression in breast tumor samples is needed to ascertain the utility of this protein as a predictive and prognostic biomarker.

## Abbreviations

AA: arachidonic acid
ABCC4: ATP binding cassette subfamily C member 4
ACSL4: Acyl-CoA synthetase long chain family member 4
AKT: protein kinase B
DGLA: Dihomo-γ-linolenic acid
FABP5,7: fatty acid binding protein 5,7
FADS1: fatty acid desaturase 1
LPCAT3: lysophosphatidylcholine acyltransferase 3
LPIN1: lipin1
MGLL: monoacylglycerol lipase
mTOR: mammalian target of rapamycin
PC: phosphatidylcholine
PE: phosphatidylethanolamine
PGE2: prostaglandin E2
PI3K: phosphoinositide-3-kinase
PL: phospholipid
PLA2G4: phospholipase A2 group Iva
PLIN2: perilipin 2
PTGER4: prostaglandin E receptor 4
PTGS2: prostaglandin endoperoxide synthase 2
QNBC: quadruple negative breast cancer
RPBC: receptor positive breast cancer
TAG: triacylglycerol
TNBC: triple negative breast cancer
