# Addendum: ACSL4: biomarker, mediator and target in quadruple negative breast cancer

**DOI:** 10.18632/oncotarget.28482

**Published:** 2023-08-10

**Authors:** Marie E. Monaco

**Affiliations:** ^1^Department of Neuroscience and Physiology, NYU Grossman School of Medicine, New York, NY 10016, USA; ^2^VA NY Harbor Healthcare System, New York, NY 10010, USA

**This article has an addendum:** No external funding was obtained with respect to this publication.

Original article: Oncotarget. 2023; 14:563–575. 563-575. https://doi.org/10.18632/oncotarget.28453

